# Effect of Acetone as Co-Solvent on Fabrication of Polyacrylonitrile Ultrafiltration Membranes by Non-Solvent Induced Phase Separation

**DOI:** 10.3390/polym14214603

**Published:** 2022-10-29

**Authors:** Alexey Yushkin, Andrey Basko, Alexey Balynin, Mikhail Efimov, Tatyana Lebedeva, Anna Ilyasova, Konstantin Pochivalov, Alexey Volkov

**Affiliations:** 1 A.V. Topchiev Institute of Petrochemical Synthesis RAS, 29 Leninsky Prospekt, 119991 Moscow, Russia; 2 G.A. Krestov Institute of Solution Chemistry of the Russian Academy of Sciences, 1 ul. Akademicheskaja, 153045 Ivanovo, Russia

**Keywords:** polyacrylonitrile, NIPS, acetone, co-solvent, pore size, MWCO, membrane, ultrafiltration, phase separation, ternary phase diagram

## Abstract

For the first time, the presence of acetone in the casting solutions of polyacrylonitrile (PAN) in dimethylsulfoxide or N-methyl-2-pyrrolidone was studied with regards to thermodynamical aspects of phase separation of polymeric solutions induced by contact with non-solvent (water), formation and performance of porous membranes of ultrafiltration range. The positions of the liquid equilibrium binodals on the phase diagrams of these three-component and pseudo-three-component mixtures were determined. For PAN—N-methyl-2-pyrrolidone—water glass transition curve on a ternary phase diagram was plotted experimentally for the first time. The real-time evolution of the structure of mixtures of PAN with solvents (co-solvents) upon contact with a non-solvent (water) has been studied. The thermodynamic analysis of the phase diagrams of these mixtures, together with optical data, made it possible to propose a mechanism of structure formation during non-solvent induced phase separation of different mixtures. The addition of acetone promotes the formation of a spongy layer on the membrane surface, which decreases the probability of defect formation on the membrane surface and keeps finger-like macrovoids from the underlying layers of the membrane. It was shown that the molecular weight cut-off (MWCO) of the membranes can be improved from 58 down to 1.8 kg/mol by changing the acetone content, while polymer concentration remained the same.

## 1. Introduction

Polymeric membranes are widely used in the separation of various liquid streams due to their lower environmental impact and investment costs with higher energy efficiency [1]. Different polymers are used for the fabrication of filtration membranes including polysulfone [2], polyethersulfone [3], cellulose acetate [4], poly(vinylidene fluoride) [5], polypropylene [6], polyacrylonitrile [7] and others [8,9,10]. Polyacrylonitrile (PAN) is a widely used material for the fabrication of membranes for aqueous and organic liquid separation because of its good mechanical and film-forming properties, stability in solvents such as hydrocarbons, alcohols, or mild aprotic solvents, good fouling resistance, but also low cost [11]. Since PAN is a barrier material towards liquid transport, the filtration membranes made of PAN can be prepared by the non-solvent induced phase separation (NIPS) method, and the typical solvents for PAN are N,N-dimethylformamide (DMF), N-methyl-2-pyrrolidone (NMP) or dimethylsulfoxide (DMSO) [7]. The porous structure of a membrane is formed during the polymer precipitation from its solution cast as a thin layer by contact with a non-solvent (coagulation bath).

Good solvent resistance of neat PAN membranes to various organic solvents results from the intermolecular dipole-dipole interactions of the nitrile side groups, and the absence of other functional groups in the polymer backbone. However, the biggest drawback of PAN is the control of pore sizes in the resulting membranes, especially decreasing the pore size by achieving the molecular weight cut-off (MWCO) lower than 5–8 kg/mol.

The membrane structure and pore size distribution are typically regulated by the adjustment of casting solution and coagulation bath compositions, including the addition of other components. In the case of PAN, polyethylene glycol (PEG) [12], polyvinyl pyrrolidone [13], or salts [14] are usually used for membrane preparation. The addition of metal-organic frameworks [15,16] also can be used to improve membrane performance, but in the case of PAN, this method is less widespread.

On the other hand, the decrease of pore size in the membrane can be achieved by the addition of more volatile solvents such as acetone, 1,4-dioxane, or tetrahydrofuran (THF) [17] that have a boiling temperature lower than aprotic solvents used for polymer dissolution. As a result, the partial evaporation of this volatile co-solvent before the immersion in the coagulation bath can increase the polymer concentration in the subsurface area, resulting in the formation of a denser top layer. Since the NIPS process is mainly governed by the diffusion and transport of components in and between two phases (polymeric solution and coagulation bath), the mobility of components can play a role in the formation of the porous structure. For instance, if the solvent molecules diffuse faster from the polymeric solution to the non-solvent phase rather than the non-solvent molecules to the polymeric solution, a membrane with a smaller pore size and a denser skin layer is expected to be formed [10]. Thus, low-volatility co-solvents are used to reduce the pore size of membranes. In particular, we recently showed [18] that the addition of acetone as a co-solvent in a casting solution resulted in a decrease in the pore size of PAN membranes prepared by the vapor-induced phase separation (VIPS) method. In the case of PAN solutions with a volatile component, the casting process involves the displacement of a mild precipitant (for instance acetone) with a harsh precipitant (water).

The thermodynamic aspects of the membrane formation process by the NIPS method are usually considered using a ternary phase diagram with consideration of evaluation of polymer-solvent-non-solvent system during the phase inversion described earlier by C.A.Smolders [19,20,21,22,23], A.M.W.Bulte [24,25,26], and others [27,28]. The polymer precipitation resulting in the formation of a porous membrane can take place via two different routes: delayed phase separation (I), and instantaneous demixing (II) [22,27,28,29,30].

I. If the solvent diffusion rate from the polymeric solution is higher than the non-solvent diffusion rate into the polymeric solution, the concentration of solvent in the thin polymeric layer near the interface is depleted, causing its densification. The further membrane formation process depends on which components have greater mass-transfer resistance through the formed skin polymeric layer. For instance, if this formed layer limits the diffusion of the solvent molecules from the bulk of the polymeric solution rather than the non-solvent molecules from the coagulation bath, then the increase of non-solvent in the sub-surface layer would drive faster the polymeric solution from its equilibria. Since the three-component system would transit through the binodal, the phase inversion takes place with the formation of two phases. The polymer-enriched phase solidifies into the randomly distributed porous structure. However, if the formed surface layer limits the diffusion of the non-solvent, then the increase of polymeric concentration would be caused by the diffusion of the solvent to the coagulation bath, which leads to the formation of denser symmetrical membranes with small pore sizes.

II. If the non-solvent diffusion rate into the polymeric solution is much higher than the solvent diffusion rate to the coagulation bath, then the decrease of thermodynamic compatibility of components of the polymeric solution leads to instantaneous demixing in accordance with the liquid–liquid mechanism.

In order to predict or describe the porous structure formation mechanism the concepts of the so-called composition paths can be used [22,27,28,31,32]. The main idea of this concept is to describe how the overall composition within the formed film or fiber depends on the time after immersion in the non-solvent. The models for the calculation of composition paths were proposed, for example, in [21,33]. They were developed based on the phase diagrams containing either only one line—the liquid equilibrium binodal (or its fragment in the region of compositions depleted in the polymer) [19,20,28,33] or, in some cases, also the spinodal [22,30,31,32]. In some works [27,28,30,34,35,36,37], the additional line representing the polymer glass transition was also plotted. The glass transition boundary is usually plotted as a horizontal line, which means that at a constant temperature the polymer can reach the glass transition at its certain threshold concentration regardless of the ratio of the other two low molecular weight components. However, it is well-known that the glass transition temperature (*T_g_*) of a polymer depends on the thermodynamic quality of the solvent (co-solvent) [38]; thus, this line cannot be a horizontal one in the ternary phase diagram.

The formation of finger-like macrovoids can compromise the mechanical characteristics of membranes, and also lead to the formation of defects in the selective layer. Different methods have been proposed in recent years to exclude the formation of such pores; for example, adding a solvent to the non-solvent [39,40], increasing the polymer concentration in the initial solution [41,42], the selecting of a poorly miscible solvent–non-solvent pair [42], an increase in the molecular weight of the polymer [43], etc. [44]. Despite this, the fundamental question of the reasons for the formation of macrovoids remains under investigation [45,46,47]. One approach to investigating it is to observe the NIPS process directly. A technique providing such an observation was proposed as early as 1972 [48], but since then it has been used only in a small number of works [9,39,40,49,50,51,52], although a new version of this technique has recently been proposed to increase the correctness of the observed structures [9,10]. At the same time, this technique has not been used to elucidate the mechanism of the structure formation in PAN membranes.

Thus, this work aims to study the phase equilibrium in mixtures of PAN with DMSO, NMP, and their mixtures with acetone, including the experimental determination of a polymer glass transition line on the ternary phase diagram. The visualization of the structure evolution of these mixtures during the polymer precipitation was used to highlight the role of the acetone on the morphology and transport properties of resulted PAN membranes.

## 2. Materials and Methods

### 2.1. Materials

Acrylonitrile monomer (>99.5) was purchased from Fluka (Switzerland). Dimethyl sulfoxide, DMSO (>99%), NMP (>99%), DMF (>99%), Acetone (>99%), ammonium peroxodisulfate, sodium dithionite, sulfuric acid, potassium carbonate, polyethylene glycol (PEG, 1; 2; 5 and 10 kg/mol), lysozyme (14.4 kg/mol), pepsin (34.5 kg/mol), ovalbumin (45 kg/mol) and bovine serum albumin (BSA, 69 kg/mol) were acquired from Khimmed (Russia). Helium with a purity of at least 99.95% was purchased from the Moscow Gas Refinery Plant.

The synthesis of PAN was carried out in an aqueous medium in the presence of a redox system consisting of ammonium peroxodisulfate ((NH_4_)_2_S_2_O_8_) and sodium dithionite (Na_2_S_2_O_4_) as initiators. 300 mL of bidistilled water was added to the Erlenmeyer flask. Then, sulfuric acid and monomer were added consequentially with concentrations [H_2_SO_4_] = 1.9 × 10^−2^ mol/L and [acrylonitrile] = 1.27 mol/L. The initiators were added to the flask simultaneously with concentrations [(NH_4_)_2_S_2_O_8_] = 5.88 × 10^−3^ mol/L, [Na_2_S_2_O_4_] = 2.52 × 10^−3^ mol/L. The prepared solution was shaken and placed in a thermostat for 40 min at 60 °C. 100 mL of solution of sulfuric acid [H_2_SO_4_] = 1.9 × 10^–2^ mol/L and monomer [acrylonitrile] = 0.66 mol/L was added. The reaction then continued for 4 h. The polymer was filtered, then washed sequentially in a solution of potassium carbonate in water and methanol to remove sulfuric acid, and dried under vacuum to constant weight. The polymer yield was 92.4%.

The study of the molecular weight characteristics of the resulting polymer was carried out by gel permeation chromatography (GPC) on a GPC-120 chromatograph (PolymerLabs). The analysis was carried out at 50 °C in DMF. The average molecular weight of the synthesized PAN (M_w_) was 118,800 g/mol. The obtained polymer was characterized by a polydispersity index (M_w_/M_n_) of 3.2.

### 2.2. Phase Diagrams for Three- and Pseudo-Three-Component Mixtures of PAN with a Solvent (Co-Solvent) and a Non-Solvent

The cloud point method used to construct phase diagrams. Weighed portions of PAN and a known mass of solvent placed in a bottle with a magnetic stirrer bar. Then the bottle was closed and placed on a magnetic stirrer with a surface temperature of 60 °C. The two-component (or the pseudo-two-component) system stirred until a homogeneous optically transparent mixture of components was formed. In all cases, the duration of stirring was 8 h. The resulting homogeneous mixture of components was cooled to room temperature and kept at this temperature for 14 h. The samples were stored in a closed container, which made it possible to minimize the evaporation of volatile components. Then, after making sure that the mixture remained optically transparent, water was carefully added dropwise. Distilled water was used as a non-solvent, and pure DMSO, NMP, as well as their mixtures with acetone (co-solvent) were used as solvents at different ratios of components.

The addition of water continued until the appearance of stable turbidity of the mixture throughout the volume. To determine the coordinate of a point on the phase diagram, the amount of each component in the opalescent mixture was estimated by weighing it on an analytical balance.

### 2.3. Determination of the Glass Transition Temperature of PAN in the Absence and Presence of Solvents (Co-Solvents)

The samples of PAN mixtures with NMP were used in differential scanning calorimetry (DSC) experiments prepared according to the following procedure. 30 wt.% of PAN and 70 wt.% of NMP were placed into a hermetically sealed flask and heated at a temperature of 100 °C for 8 h until homogeneous mixtures were obtained. Then, the flask was opened and placed in a vacuum oven (T = 100 °C; p = 10 mmHg) for partial evaporation of NMP to obtain mixtures with the required polymer concentration. The polymer concentration was evaluated by periodically weighing the flask with samples on an analytical balance. To obtain three-component mixtures (PAN, NMP, water), a binary mixture was placed in a closed bottle above the water surface at a temperature of 80 °C to dissolve water vapor in the sample. The mass of water dissolved in the sample and the ratio of components in the resulting three-component mixture were evaluated by weighing on an analytical balance. Keeping the samples above the water surface was carried out for a time that ensures the achievement of the required ratio of components in the mixture.

The glass transition temperature was determined using the DSC method. Thermograms of PAN and its mixtures with solvents of various compositions were obtained on a NETZSCH 204F1 Phoenix calorimeter (scanning rate 10 °C/min, the weight of samples in pressed crucibles 3–7 mg, standard calibration). DSC experiments were carried out according to the following scheme: cooling down to −40 °C, heating up to 120 °C (in the case of PAN–NMP binary mixtures) or up to 80 °C (in the case of mixtures containing water), holding at this temperature for 5 min, and re-cooling down to −40 °C.

### 2.4. The Evolution of the Structure of PAN Mixtures with Various Solvents (Co-Solvents) in Contact with Water

The evolution of the structure of PAN mixtures with various solvents (co-solvents) in contact with water was investigated by optical observation with a microscope Mikromed C-11. A preliminarily prepared homogeneous mixture of PAN with a solvent (co-solvent) of the required composition was placed in the form of a droplet with a volume of ~40 μL on glass with a bounding frame. The bounding frame is made of two parallel strips of the polyimide film 50 µm thick. The distance between the strips is ~30 mm, and their length is ~90 mm.

**Figure 1:**
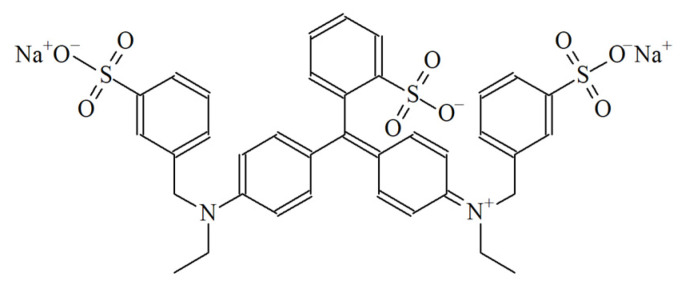
The chemical formulae of E133 Dye.

The lower glass with a drop of the mixture located in the center of the frame was covered with another glass. The glass was fixed with spring clips and transferred to the working table of the optical microscope. Then, water colored with the E133 dye (Figure 1), was fed through the syringe needle. Due to the capillary effect, water filled the entire space between the glasses limited by the frame. This design of the “experimental cell” made it possible to achieve a bath modulus of ~1:50, which is comparable to the bath modulus in the actual process of membrane formation. Events that occur after contact with a mixture drop with water were recorded using a digital camera.

### 2.5. Membrane Preparation and Characterization

The membranes were prepared by the NIPS method with water as a non-solvent. The polymer solution was cast on a glass plate with a thickness of 200 μm with a casting speed of 2.5 m/min. The membrane casting was carried out at 20 °C and 20% humidity. The cast film was immediately immersed in the coagulation bath (distilled water, 20 °C) for 24 h. After completion of membrane formation, the samples were washed with distilled water and kept in a new portion of distilled water for 24 h to wash out the solvent residues. The water was then replaced with fresh water for further storage of the obtained membranes until use.

Filtration experiments were carried out in a dead-end stirred filtration cell. At least three membrane coupons were measured, and the recorded data were averaged. For filtration experiments, the membrane coupon was placed onto the porous stainless steel disks and sealed with a rubber O-ring. The active membrane area in the cell was 7.9 cm^2^. The system was pressurized with helium. The transmembrane pressure was 5 bar. Membrane permeance was measured from the beginning of the experiment without initial pre-pressurization. Permeate samples were taken every 5 min until constant permeance was obtained for 5 or more data points. Sample permeance was determined as an average for the last 5 data points. In the case of solution filtration, the feed was stirred at 550 rpm to minimize the concentration polarization effect. The membrane permeance *L_p_* (L/(m^2^·h·bar)) was determined as:

(1) LP=JΔp=mρ·S·t·Δp,

where *J* is the liquid flux, ∆*p* is the trans-membrane pressure (bar), *m* is the mass of the permeate (g), *ρ* is the density of the permeate (g/L), *S* is the active membrane area (m^2^), and *t* is the filtration time (h).

Raw solutes were selected for separation performance characterization, namely PEG, lysozyme, pepsin, ovalbumin, and BSA. PEG had molecular weights of 1, 2, 5, and 10 kg/mol. A new membrane coupon was used for every rejection experiment. Before the rejection test, distilled water was filtered through a membrane coupon for 1 h at 5 bar. The separation test was carried out at the transmembrane pressure of 5 bar for at least 2 h to achieve steady-state values of the rejection. Phosphate buffer (0.1 M) with pH 7.0 was used for BSA solution preparation. Other proteins and PEG were dissolved in distillate water. All solutions were prepared with a solute concentration of 0.5 g/L. The concentration of proteins in the feed and permeate was measured with a spectrophotometer at the wavelength of 280 nm. PEG content was determined by the weight method after the evaporation of solvent at 80 °C to constant weight. The rejection *R* was calculated using the relation:

(2) R=1−CpCf·100%,

where *C_f_* and *C_p_* denote the solute concentrations in the feed and permeate, respectively.

The pore size was measured by liquid–liquid displacement porosimetry using the porometer POROLIQ 1000 ML (Porometer, Belgium). The operating principle is based on the measurement of the equilibrium pressure corresponding to the flux of the displacing liquid. The displacement of the wetting liquid was carried out by a stepwise increase of the transmembrane pressure with monitoring of the flux through the membrane after 180 s initial stabilization time at each applied pressure. The measurement was stopped after reaching a linear dependence of the flux on pressure, which indicated a complete displacement of the wetting liquid. The alcohol-rich phase was used as the wetting liquid and the water-rich phase was used as the displacing liquid. 3 coupons (2 cm in diameter) were cut from every membrane and were placed into the beaker with the wetting liquid for at least 2 h at 20 °C before the testing. The results were averaged for all investigated samples. The measurements were carried out at 25 °C by using a pair of immiscible liquids prepared by demixing a mixture of isobutanol and water (1/4, *v*/*v*). The diameter (*D*) of the open pore is related to the trans-membrane pressure via the Young–Laplace equation:

(3) D=4·γ·cosθΔp,

where *γ* is the interfacial tension between the two liquids, *θ* is the contact angle between the membrane and the wetting liquid (complete wetting is assumed, i.e., cos*θ* = 1), and Δ*p* is the trans-membrane pressure. Interfacial tension *γ* for the mixture of isobutanol and water is 1.9 mN/m at 25 °C. Pore size was calculated according to the procedure described in detail in [7].

Scanning electron microscopy (SEM) was used to characterize the structure and morphology of the membranes. SEM was carried out on a Thermo Fisher Phenom XL G2 Desktop SEM (USA). Cross-sections of the membranes were obtained in liquid nitrogen after preliminary impregnation of the specimens in isopropanol. A thin (5–10 nm) gold layer was deposited on the prepared samples in a vacuum chamber (~0.01 mbar) using a desktop magnetron sputter “Cressington 108 auto Sputter Coater” (UK). The accelerating voltage during image acquisition was 15 keV.

## 3. Results

### 3.1. Ternary Phase Diagrams of Three-Component Mixtures and Pseudo-Three-Component Mixtures

For rapid assessment of the thermodynamic affinity between a polymer and a low molecular weight (LM) substance, Hansen’s solubility parameters were used [53]. The distance in the Hansen space between the polymer and the LM substance was calculated using the following relation:

(4) $$R_{P-S}=\sqrt{4\cdot {\left(\delta_{d,P}-\delta_{d,S}\right)}^{2}+{\left(\delta_{p,P}-\delta_{p,S}\right)}^{2}+{\left(\delta_{h,P}-\delta_{h,S}\right)}^{2},}$$

where *δ_d_*, *δ_p_* and *δ_h_* are the Hansen solubility parameters, reflecting the dispersion, polar interaction, and hydrogen bonds, respectively, and the *P* and *S* indexes correspond to polymer and solvent. Solubility parameters for two- and three-component mixtures are calculated as the sum of the individual solubility parameters of components multiplied by their molar concentration. In Hansen’s space criterion of polymers, solubility is the inequality of *R_P-S_ ≤ R*_0_, where *R*_0_ as the solubility radius for PAN is 10.9 MPa^0.5^ [53].

**Figure 2:**
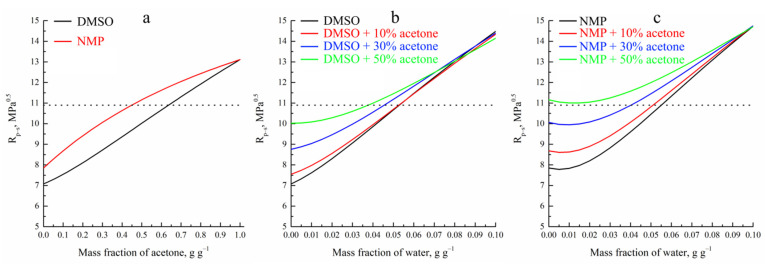
The distance in Hansen space between PAN and binary mixtures of DMSO and NMP with acetone (**a**); binary or pseudobinary mixtures of DMSO (DMSO + acetone) (**b**) and NMP (NMP + acetone) (**c**) with water. A dashed horizontal line corresponds to PAN solubility radius.

Figure 2a shows the dependences of the calculated *R_P-S_* values for the mixtures of DMSO and NMP with acetone from the mass fraction of the former. The dependences of the *R_P-S_* for ternary mixtures of water and acetone with DMSO and NMP from the amount of water are illustrated in Figure 2b,c respectively. One can see that DMSO and NMP are good solvents, whereas acetone is a poor solvent of PAN. DMSO and its mixture with acetone have a higher thermodynamic affinity to this polymer than NMP. From the comparison of the intersection points of the curves given in this figure with a horizontal line, corresponding to the value of *R*_0_, it was found that more acetone can be added to DMSO than to NMP with maintaining PAN solubility

Figure 2 shows that with an increase of acetone amount in a mixed solvent for both DMSO and NMP, the amount of water that transforms three-component mixtures into a non-solvent for PAN, i.e., that makes *R_P-S_* larger than *R*_0_, is reduced.

Taking into account the data on the Hansen solubility parameters, three compositions with every solvent were chosen for further investigation. At first, binary mixtures of PAN with DMSO or NMP without acetone were applied. The second compositions are pseudo-binary mixtures of PAN with mixed solvents consisting of DMSO or NMP and a small amount (10 wt.%) of acetone. The later composition is different for different solvents—pseudo-binary mixtures of PAN with co-solvents consisting of DMSO or NMP and the maximum amount of acetone, which retains the possibility of preparing a homogeneous solution at room temperature in a reasonable time. In the case of DMSO, 50 wt.% of acetone was used, whereas for NMP, maximum acetone content was 30 wt.%.

**Figure 3:**
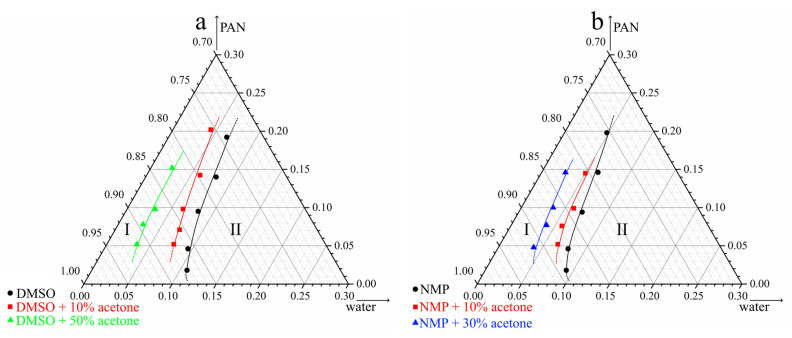
Ternary phase diagrams of mixtures PAN—DMSO (DMSO + acetone)—water (**a**) and PAN—NMP (NMP + acetone)—water (**b**).

Figure 3 shows the ternary phase diagrams of (PAN, DMSO, water and PAN, NMP, water) and pseudo-three-component (PAN, DMSO + acetone, water and PAN, NMP + acetone, water) mixtures containing various amounts of acetone. All phase diagrams are of the same type and contain a single boundary line—the liquid equilibrium binodal, which limits two regions. I—a single-phase region in which homogeneous three- or four-component mixtures exist and II—a two-phase region in which a solution of polymer in a mixture of two or three-component liquids (phase 1) coexists with a solution of such mixtures of liquids in a polymer (phase 2). The compositions of the first and second coexisting phases are given by the lower and upper parts of the binodal.

From a comparison of the position of the curves in the concentration field of the phase diagrams, it follows that an increase in the mass fraction of acetone added to DMSO or NMP is accompanied by a shift in the liquid equilibrium binodal towards compositions depleted in water (to the left). This means that as the amount of acetone in both co-solvents increases, their thermodynamic affinity for the polymer deteriorates, and the phase separation of homogeneous mixtures takes place with a smaller amount of water.

It can also be seen, that the thermodynamic affinity of NMP and its mixtures with acetone for the polymer is lower than that of DMSO and its mixtures with acetone. The conclusions following from the analysis of phase diagrams are in good agreement with the conclusions made above when discussing the solubility parameters characterized by *R_P-S_* values.

### 3.2. Influence of Three-Component Mixture Composition on Glass Transition Temperature

**Figure 4:**
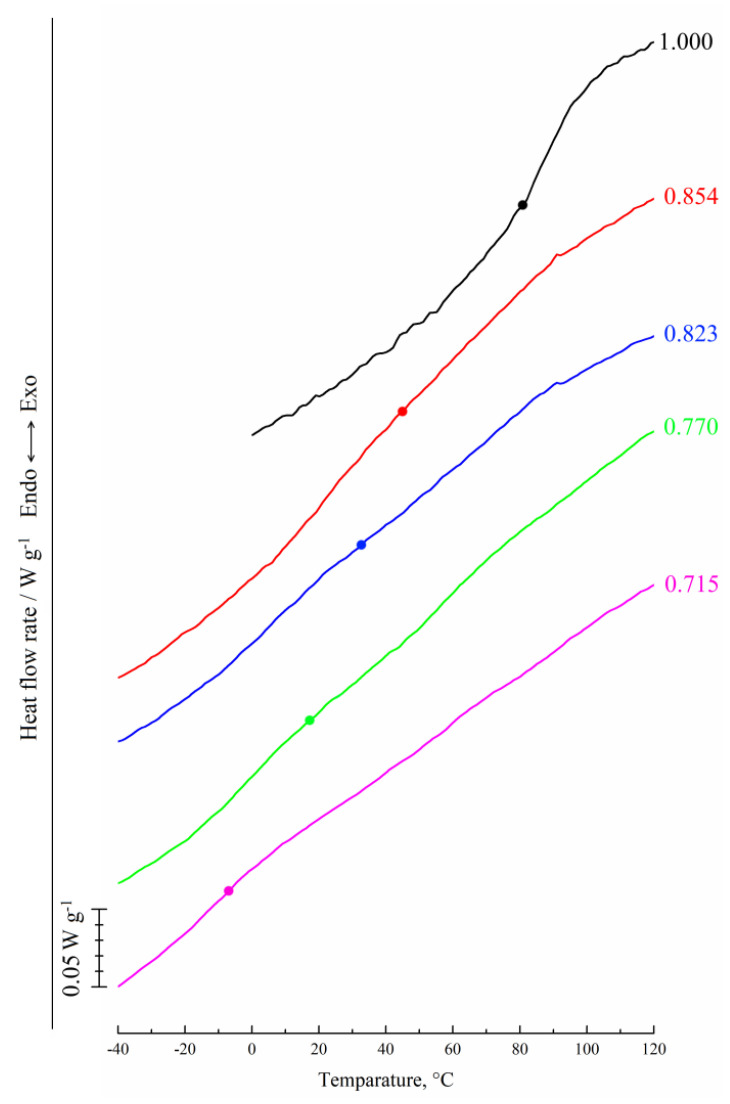
DSC thermograms obtained in the cooling mode for mixtures of PAN with NMP of various compositions. The mass fraction of the polymer in the mixture is indicated to the right of the curves.

The glass transition line in the ternary phase diagram reflects the dependence of the polymer concentration in a multicomponent system on the ratio of components in a mixture. To plot the glass transition line in the ternary phase diagram, DSC experiments were performed with PAN and its mixtures with NMP and with co-solvents consisting of NMP and various amounts of water. Since we are considering the compositions at which the polymer passes from a viscous (highly elastic) to a glassy state, DSC thermograms were obtained in the cooling mode. Since all the curves obtained for different solvents are similar, further discussion will be done using DSC thermograms recorded for mixtures of PAN with NMP of various compositions (Figure 4).

**Figure 5:**
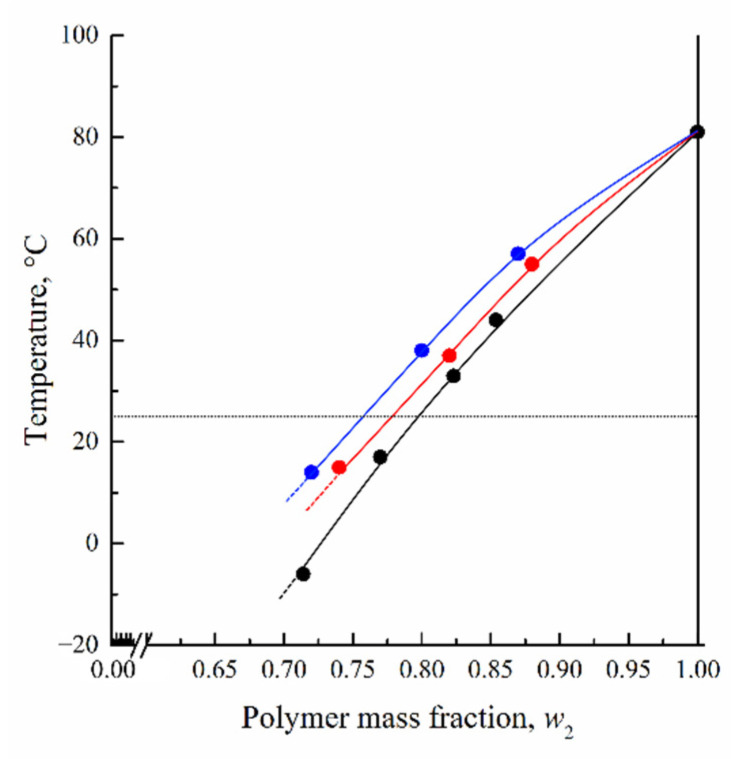
Dependences of PAN *T_g_* on the composition of the binary mixture of PAN with NMP (black dots) and pseudobinary mixtures consisting of PAN and mixtures of NMP with water, containing 5 (red dots) and 10 (blue dots) wt.%, determined by DSC in the cooling mode.

**Figure 6:**
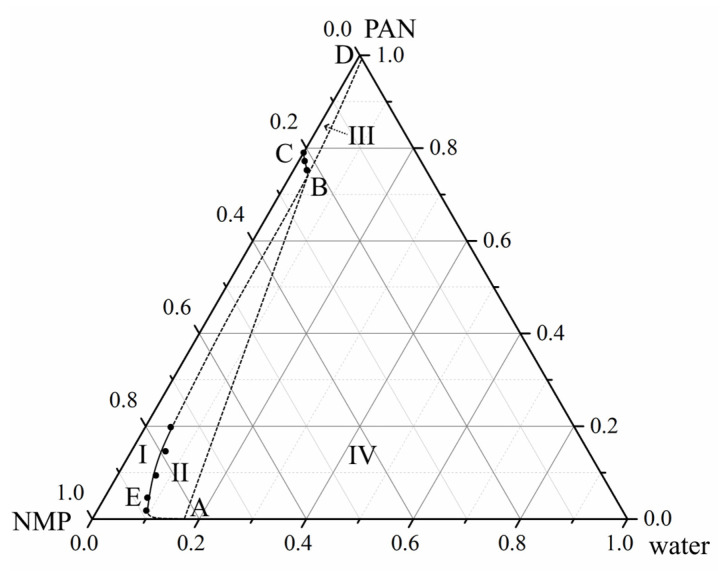
A ternary phase diagram of a mixture of PAN with NMP and water.

In cooling mode, the temperature *T_g_* corresponding to the middle of the glass transition step decreases with an increase in the amount of NMP in the mixture with PAN from 0.146 to 0.285 *g*/*g*. At a higher NMP content in the mixture, glass transition was not observed. Similar tendencies were also observed for a ternary mixture of PAN with NMP and water of various compositions. The comparison of the glass transition temperature curves at different water content reveals that *T_g_* for PAN decreases as a result of NMP presence while the addition of water reduces this effect (Figure 5). The coordinates of the intersection of these curves with the horizontal isotherm corresponding to T = 25 °C were used to plot the BC fragment of the polymer glass transition line on the ternary phase diagram for PAN mixtures with NMP and water (Figure 6).

The BC line in Figure 6 has a significant slope and is not parallel to the axis of the solvent-non-solvent compositions as shown in the schematic diagrams in [27,28,30,34,35,36,37]. The slope of the BC line indicates that with an increase in the amount of water in a three-component mixture, the plasticizing ability, which leads to the transition of the polymer from a glassy to a viscous/highly elastic state, decreases. In other words, the BC line shows how the ratio between the solvent and the non-solvent must change with an increase or decrease of the polymer concentration in a three-component mixture in order for the polymer to remain in a glassy state. Hypothetical dashed lines plotted on this diagram correspond to the continuation of the liquid equilibrium binodal, including in the region of the glassy state of the polymer (segment BD) and part of the glass transition line AB. The lines shown in the phase diagram have the following thermodynamic meaning. Curve AEB is a liquid equilibrium binodal. Line ABC reflects the glass transition boundary of PAN in three-component mixtures of various compositions. Note that in section AB the composition of the mixture that vitrifies is always equal to the composition corresponding to point B. BD curve reflects the dependence of the swelling degree of glassy PAN on the ratio of NMP and water in their mixture. These lines limit four areas on the phase diagram:

I—a single-phase region in which there are homogeneous molecular mixtures of a polymer with a solvent and a non-solvent;

II—a two-phase region in which polymer solutions in a mixture of two LM liquids and solutions of this mixture in a polymer coexist;

III—a single-phase region, in which there is a solution of a mixture of two LM liquids in a glassy polymer;

IV—a two-phase region in which a solution of a mixture of two LM liquids in a glassy polymer and a mixture of two LM liquids coexist;

The diagram, supplemented by the indicated boundary lines, makes it possible to characterize the state of a three-component system at any point in the concentration field. It should be noted that most of the phase diagrams for the amorphous polymer—solvent—non-solvent mixtures, including those studied in this work, will have a similar topology. Since the construction of this line is a rather labor-intensive process, researchers usually avoid this part of the diagram. However, it was important to show what the phase diagram looks like for such systems.

The topological analysis of the extended phase diagram permits to improve understanding of the evolution of the structure of homogeneous mixtures of PAN with NMP of various compositions when immersed in water at room temperature. When the initial binary mixture of PAN with NMP is immersed in the precipitator, the mutual diffusion of water into this mixture and NMP from it into water occurs due to the high thermodynamic affinity between the diffusing components. As a result, a homogeneous three-component mixture of PAN with NMP and water is formed. As these mass transfer processes continue, the composition of a homogeneous three-component system first reaches values corresponding to the point belonging to the liquid equilibrium binodal AEB (Figure 6), and then its microphase decomposition is realized with the formation of an emulsion consisting of two phases: enriched and depleted in the polymer. The compositions of these phases changes with a change in the thermodynamic affinity of a two-component liquid, which depends on the ratio of NMP and water, with respect to the polymer, according to the binodal branches AE and EB.

When, in the course of the above mass transfer processes and microphase decomposition, the polymer-enriched phase reaches the composition corresponding to point B (Figure 6), the glass transition of the polymer occurs in the presence of NMP and water dissolved in it, and the morphology of the resulting emulsion fixes.

According to the obtained phase diagram, at room temperature, the glass transition occurs when the mixture consists of 73.8 wt.% of PAN, 22.7 wt.% of NMP, and 3.5 wt.% of water (Point B in Figure 6). From a practical point of view, the discussed phase diagram allows us to predict the range of solution compositions, which ensures the formation of a spongy porous structure by the NIPS method. At the same time, to elucidate the features of the formed porous structures, it is necessary to carry out additional experiments with measures of the mass transfer rates between the solvent and the non-solvent.

### 3.3. Structure Formation in PAN/Solvent Systems and Its Mixtures with Acetone after Immersion in Water

The structure formation on the PAN mixtures was investigated using an optical microscope with continuous image recording for a time period that ensures the advancement of the front of structure formation in the radial direction from the border of the sample (droplet) to its center. The compositions of the investigated mixtures are listed in Table 1.

**Figure 7:**
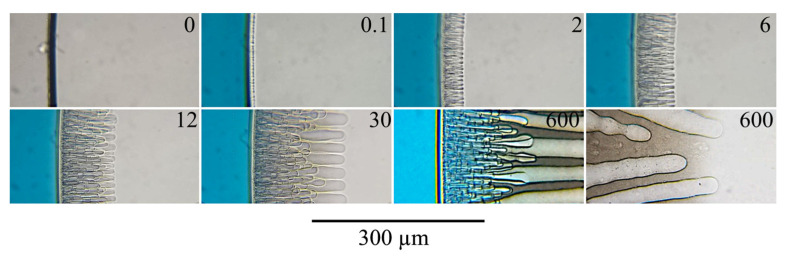
The evolution of the structure of a mixture of PAN with DMSO containing 15 wt.% of polymer in contact with water. The time in seconds from the moment of contact between the sample and the non-solvent is indicated in the upper right corner of each photo.

Real-time optical observations of the mixture of PAN with DMSO after contact with water (Figure 7) shows that after the contact of the mixture with the non-solvent, it takes 0.1 s for an inhomogeneity to appear in the surface layer of the sample, and its boundary moves towards the center of the drop. After mixture-non-solvent contact, two mass transfer processes occur the extraction of the solvent into the non-solvent and the penetration of the non-solvent into the sample. The displacement of the interface means that due to the aforementioned processes, the size of the sample decreases, which, obviously, is possible only if the rate of the first process exceeds the rate of the second. As a result, the surface layer becomes highly enriched in the polymer. It begins to act as an obstacle to mass transfer between the inner layers of the sample and the non-solvent. At the same time, the surface layer is enriched with the non-solvent until its composition reaches the liquid equilibrium binodal (section EB in Figure 6). When this composition is obtained, liquid decomposition begins in the surface layer and both closed and open pores are formed in it, filled with a mixture of solvent and non-solvent. The diffusion of DMSO and water through the open pores proceeds much faster than through the surrounding matrix enriched with polymer. Water mass transfer through open pores leads to the enrichment of the next layer with the non-solvent. This process launches the formation of finger-like pores, which become clearly visible 2 s after the contact of the sample with the non-solvent. Due to the high rate of water supply, the mixture in contact with the open surface pore gains the composition corresponding to the binodal of liquid equilibrium earlier. As a result of the phase separation, relatively large droplets of a polymer-poor liquid are formed, which grow towards the center of the sample forming finger-like pores. Over time, the growth of some of the fingers slows down and then stops, and the diameter of the fingers that continue to grow increases, reaching 20 or more microns after 30 s of the observation. This increase is not monotonous—the diameter of the developing finger along its length turns out to be unstable, as is clearly visible in the last photograph in Figure 7.

It can be seen that at some distance behind the growing fingers front, another front becomes noticeable after 12 s, to the left of which, the mixture located between the fingers scatters light, and to the right, remains homogeneous. At the beginning of the process, this front was difficult to distinguish due to too many thinner fingers overlapping each other. This front can be better seen in the last photograph taken 10 min after mixture-non-solvent contact. The appearance of this front reflects the liquid–liquid microphase separation, leading to the formation of a thin emulsion of droplets of a phase depleted in the polymer in a phase enriched in it. The movement of the front towards the center of the sample indicates the continuity of the microphase separation due to the constant influx of water into the sample and the outflow of the solvent from it. Although the structure of the resulting emulsion cannot be distinguished with an optical microscope, due to the frequent alternation of phases with different refractive indices, such an emulsion scatters light and appears darker in the light. The non-solvent necessary to achieve a composition corresponding to the binodal and the subsequent liquid decomposition enters the area between the fingers both through channels in the surface layer and finger-like pores and through the “monolithic” sections of the surface layer by the diffusion mechanism.

The investigation of the sample structure after 10 min illustrates that the intensity of light scattering by the areas between the fingers increased significantly during this time. This is probably caused by two factors: an increase in the difference in the refractive indexes of the coexisting phases as their compositions change in accordance with the AE and EB binodal branches, and the coalescence of the initially formed emulsion. In addition, it can be seen that the intensity of the blue coloration of the non-solvent near the sample decreased, while the boundary layers of the sample, on the contrary, turned blue (Figure 7). This means that although the primary phase decomposition occurs relatively quickly, a further change in the formed structure, complete leaching of the solvent from the sample, and averaging of the concentration of the components in the entire volume of both the precipitation bath and the formed pores require a rather long duration. Further observation has shown that the uniform coloring of the liquid in the pores of the sample with a diameter of about 2 mm occurs ~2 h after its contact with the non-solvent. The variation in thickness of the growing finger-like pores mentioned above is associated with fluctuations in the rate of water supply to the front of their growth.

When, in the course of continuously occurring mass transfer processes and phase separation, the polymer-enriched phase reaches a composition at the glass-transition line (point B in Figure 6), the formed structure of the sample is fixed. Therefore, following the movement of the turbidity front, the next front moves, at the boundary of which the resulting porous structure is fixed. However, since the glass transition does not change the optical properties of the polymer, this layer was not detected in the experiments on an optical microscope.

**Figure 8:**
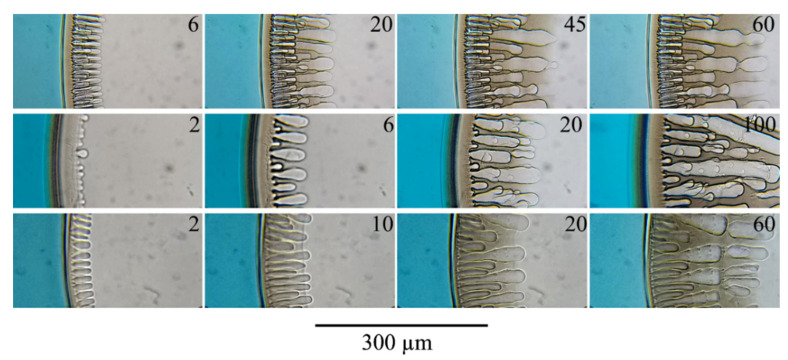
The evolution of the structure of a mixture of 15 wt.% PAN with DMSO/Acetone ratio 90/10 (Composition 2 from Table 1)—upper row; DMSO/Acetone ratio 50/50 (composition 3)—middle row; 20% PAN with DMSO (composition 4)—the bottom row. The time in seconds from the moment of contact between the sample and the non-solvent is indicated in the upper right corner of each photo.

An increase in the concentration of the polymer in the initial mixture from 15 to 20 wt.% or partial replacement of DMSO with acetone leads to only minor changes in the process of the structure formation (Figure 8). An increase in the concentration of acetone in the mixed solvent leads to an increase in the thickness of the surface layer (distance from the surface of the sample to the beginning of finger-like pores). The contact of a three-component mixture with a non-solvent (water) is accompanied by diffusion of the latter into the sample and extraction of each of the co-solvent components into the non-solvent. Moreover, the extraction rate of these components depends on their thermodynamic affinity for the non-solvent. In addition, when using a co-solvent containing acetone, the latter, being highly volatile, evaporates from the surface even before contact with the non-solvent. In such a situation, the surface layer is enriched with polymer both due to the evaporation of one of the co-solvent components (acetone) and due to the extraction of the co-solvent components into the non-solvent, i.e., at a higher rate. The combination of these mass transfer processes leads to an increase in the surface layer thickness. The polymer concentration increase in this layer leads to a decrease in the number of pores, including open channels.

Comparing the structure evolution of different casting solution compositions showed that with an increase in the amount of acetone, the number of finger-like pores per unit area of the sample decreases, and their average diameter increases. This fact is in good agreement with the assumption about the formation of fingers near the open pores in the surface layer and about a decrease in the number of the latter with an increase in the concentration of acetone in the co-solvent. The cessation of finger-like pores growth and the growth of new finger-like pores observed in experiments with acetone (Figure 8) is likely a special case of the above-mentioned fluctuations in the diameter of finger-like pores and is associated with the irregular supply of water through a sufficiently extended microphase separation layer to deeper layers of the sample.

Increasing the polymer concentration to 20 wt.% in a mixture with DMSO (lower row in Figure 8) leads only to a slight increase in the thickness of the layer formed as a result of microphase separation. As in the case considered above, an increase in polymer concentration leads to a decrease in the number of finger-like pores and growth in their average diameter, which is also associated with a decrease in the number of open channels in the surface layer. In addition, the front moving behind the growth front of finger-like pores reflecting microphase decomposition is much more clearly visible. Moreover, it can be seen that this front between the finger-like pores has a concave shape. This means that the non-solvent necessary for the start of microphase separation moves mainly through the walls of the already formed finger-like pores, whereas the water flow through the surface layer by the diffusion mechanism is much lower.

**Figure 9:**
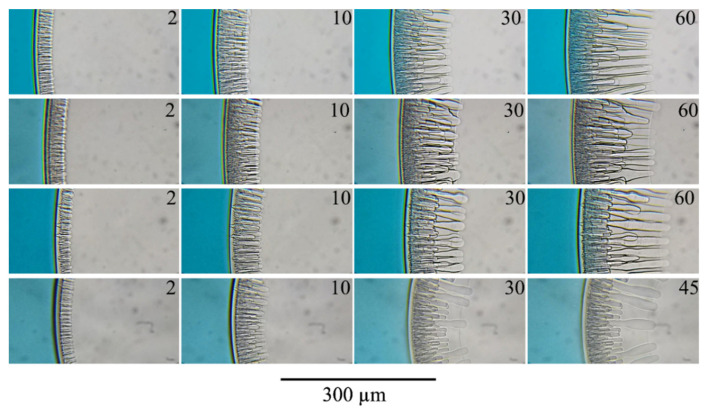
The evolution of the structure of a mixture of 15 wt.% PAN with NMP (composition 5)—upper row; 15 wt.% PAN with NMP/Acetone ratio 90/10 (Composition 6)—second row; 15 wt.% PAN with NMP/Acetone ratio 70/30 (Composition 7)—third row; 20% PAN with NMP (Composition 8)—the bottom row. The time in seconds from the moment of contact between the sample and the non-solvent is indicated in the upper right corner of each photo.

The investigation of the phase separation of liquids containing NMP as solvent (Figure 9) showed the main points about the mechanism of the structure formation made in the discussion of mixtures containing DMSO as the main solvent. Nevertheless, two peculiarities of these mixtures’ behavior can be distinguished. First, the front line of microphase liquid separation is observed on a microscope as a “bright band”. Such behavior was also revealed in [54,55]. Secondly, from a comparison of the data shown in Figure 7 and Figure 8 with image 9, it was observed that the intensity of light scattering in the matrix surrounding the finger-like pores greatly decreased in comparison with mixtures of PAN with DMSO both in the absence and in the presence of acetone. This is likely due to the fact that, as a result of the microphase separation of mixtures, smaller pores are formed, which scatter visible light less intensively.

**Figure 10:**
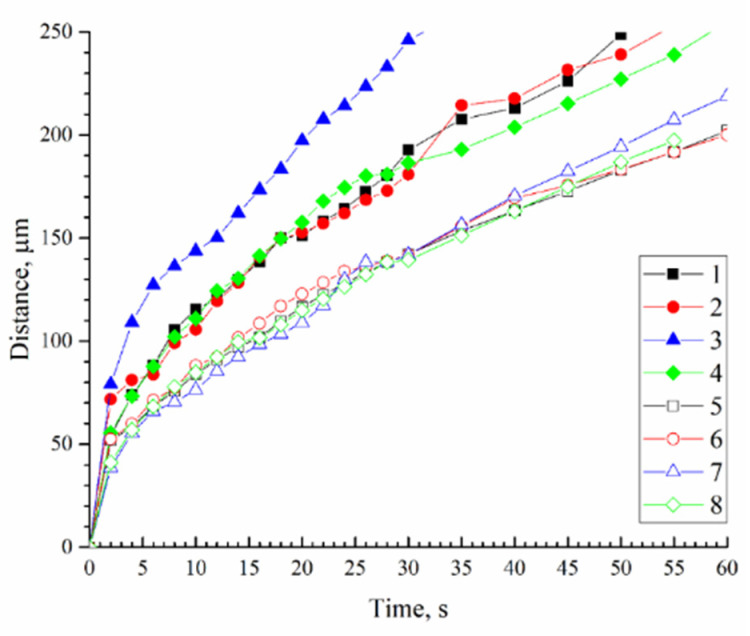
The distance by structure formation boundary from the surface with time for different casting solution compositions: 1—15 wt.% PAN with DMSO; 2—15 wt.% PAN with DMSO/Acetone ratio 90/10; 3—15 wt.% PAN with DMSO/Acetone ratio 50/50; 4—20 wt.% PAN with DMSO; 5—15 wt.% PAN with NMP; 6—15 wt.% PAN with NMP/Acetone ratio 90/10; 7—15 wt.% PAN with NMP/Acetone ratio 70/30; 8—20% PAN with NMP.

Measuring the distance that the boundary of the structural formation moves away from the surface over time shows that all systems exhibit a tendency to slow down over time (Figure 10). At the same time, the obtained curves have many local inflections, which, as the results of parallel experiments have shown, are not related to the measurement error. A comparison of these curves with video data on the evolution of the structure of mixtures allows us to conclude that the moments of an increase in the rate of structure formation correspond to an increase in the average diameter of the formed finger-like pores. Respectively, the moments of a decrease in the rate correspond to a decrease in these diameters or a complete stop in the growth of finger-like pores.

From the analysis of the obtained results, the following conclusions can be made:

1. The transition from DMSO to NMP, both in the absence and in the presence of acetone at any investigated PAN concentration, is accompanied by a decrease in the rate of the movement of the structure formation front. Taking into account that a smaller amount of water is required to transfer the initial mixture with NMP, both in the absence and in the presence of acetone (see phase diagrams in Figure 3), it can be assumed that this fact is the result of slower diffusion processes.
2. Increasing the PAN concentration from 15 to 20% wt. in mixtures containing DMSO or NMP does not affect the rate of structure formation. At the same time, Figure 3 shows that with an increase in polymer concentration lower amount of water is needed for the phase separation. This means that an increase in resistance to mass transfer due to an increase in the polymer concentration compensates for the lower water uptake needed for the start of the phase separation.
3. In the case of NMP, the addition of acetone has no effect on the rate of structure formation. On the other hand, in a mixture containing a high amount of acetone with DMSO, the speed of the structure formation front increases noticeably.

### 3.4. Morphology of the Obtained Membranes

**Figure 11:**
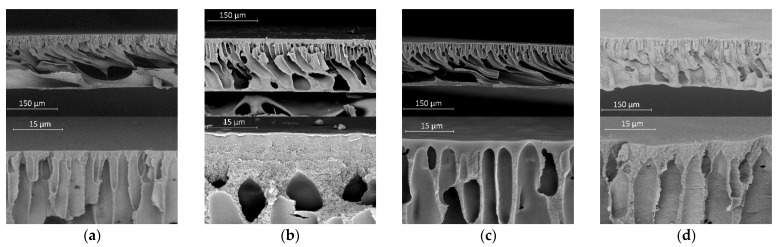
SEM images of the membrane cross-section with respect to the casting solution composition: (**a**)—15% PAN with DMSO; (**b**)—15% PAN with DMSO/Acetone 50/50; (**c**) 15% PAN with NMP; (**d**) 15% PAN with NMP/Acetone 70/30. The upper row corresponds to lower magnification (image size 550 × 350 μm); the bottom row—is a selective layer with high magnification (image size 55 × 35 μm).

The investigation of morphology for the membranes prepared by the NIPS method from different casting solutions confirms the above conclusions about the effect of the replacement of the solvent with acetone on the structure of the membranes (Figure 11). When acetone is added to the solvent (DMSO or NMP), the thickness of the surface layer and the average diameter of the fingers increase. In addition, the pores in the spongy structure of the surface layer and the walls of finger-like pores in membranes obtained from mixtures based on DMSO are noticeably larger than in membranes obtained from mixtures based on NMP. This agrees well with the conclusion made above based on data on the intensity of light scattering by a structure that has undergone microphase separation. Taking into account that the pores volume is inversely proportional to the volume fraction of the polymer in each microvolume, it can be concluded that the volumetric porosity of the surface layer has a gradient of porosity. This gradient occurred due to the mass exchange process between the casting solution and a non-solvent leading to the polymer concentration gradient in the surface layer. Therefore, the rate of all mass transfer processes, including the process of solvent extraction from the sample into the precipitation bath, was a function of not only the polymer concentration and the thermodynamic affinity of the components to each other, but also the coordinates over the sample cross-section and duration contact with the non-solvent.

### 3.5. Separation Properties of Obtained Membranes

**Figure 12:**
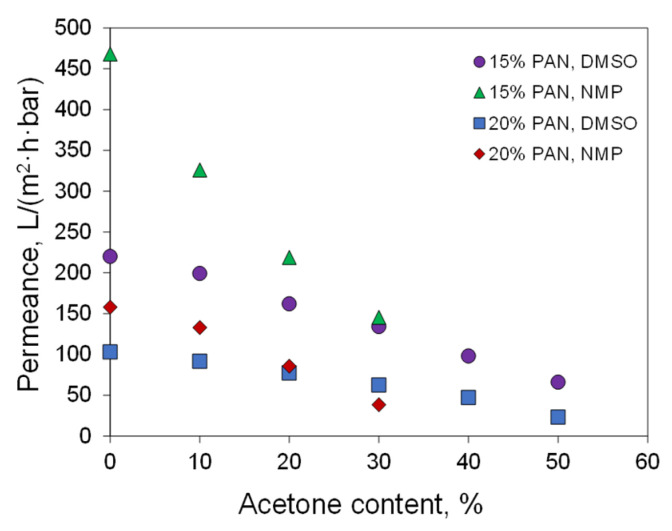
The dependence of water permeance obtained by the NIPS method on the acetone content in the casting solution.

Membranes were prepared from PAN solutions in DMSO/acetone mixtures (ratios 9/1, 8/2, 7/3, 6/4, and 1/1) and NMP/acetone (ratios 9/1, 8/2, and 7/3). The polymer concentration in the solutions was 15 and 20%. The addition of acetone decreases membrane permeance (Figure 12). In the case of 15% PAN solutions in the NMP/acetone mixture, the water permeance decreased from 468 L/(m^2^·h·bar) for membranes obtained without the addition of acetone to 145 L/(m^2^·h·bar) for the mixture NMP/Acetone 70/30. At a PAN concentration of 20%, a similar drop in permeance from 158 to 38 L/(m^2^·h·bar) was observed. For the membranes prepared using DMSO as a solvent, the decrease in permeability with an increase in acetone content is less sharp.

The main factor of the decrease in membrane permeance is the decrease in pore size. In the case of NMP, the pore size monotonically decreased from 28 and 19.6 nm for 15 and 20% PAN solutions without acetone, respectively, to 17 and 10.8 nm for the 3:7 acetone/NMP mixture (see Table 2). For the membranes from solutions with DMSO, the maximum content of acetone was higher compared to NMP, and a more significant reduction in the pore size was observed. Pore sizes of 12.8 nm and 3.7 nm were observed for the membrane from 15 and 20% PAN solutions in 1:1 acetone/DMSO, respectively. Thus, the addition of acetone to the spinning dope solution leads to a significant decrease in the pore size of the membranes, and an increase in the proportion of acetone leads to a monotonic decrease in permeability.

**Figure 13:**
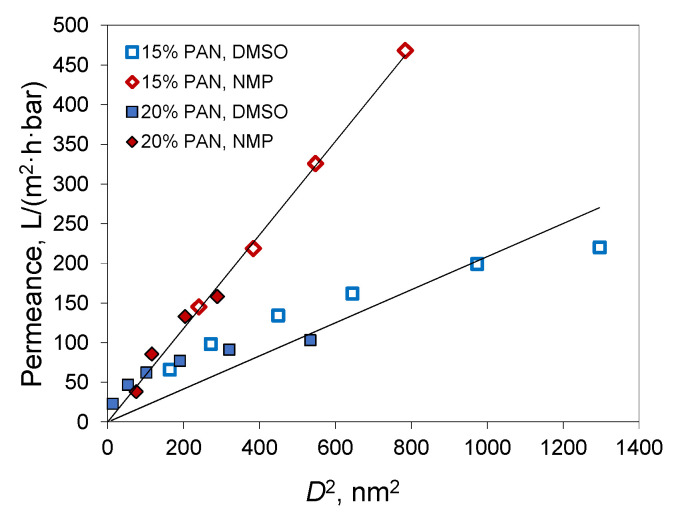
The plot of membrane permeance versus pore diameter (*D*) squared.

As mentioned, a decrease in membrane pore size is a major factor in the drop in membrane permeance. The most widely used model for the description of the flux behavior through porous membranes is the Hagen–Poiseuille equation for viscous flow [56]. Despite the fact that this equation considers parallel cylindrical pores, which is not the case for the obtained membranes, some points of this model can be used to analyze the obtained results. According to the Hagen–Poiseuille equation, membrane permeance is proportional to pore diameter (*D*) squared. This is correct for the membranes obtained with NMP (Figure 13).

For the membranes prepared from DMSO solutions, non-linear dependence of permeance on the pore diameter squared was observed. Adding acetone into the PAN solution in DMSO decreases pore size, whereas the permeance decreases not so much. No such effect is observed for NMP. This means that pore size is not the only parameter that changes in membranes when acetone is added to the casting solution. In the case of DMSO, the addition of acetone changes the parameters of the phase separation. It promotes the formation of a more permeable structure of the surface layer. This is in agreement with the appearance of a spongy layer on the membrane surface. It also correlates with the increase in speed of the structure formation front at high acetone content. Thus, the presence of acetone in the casting solution decreases the pore size and changes the structure of the membrane top layer but keeps the finger-like macrovoids from the underlying membrane layers.

From Figure 13 and Table 2 it can be concluded that NMP produces membranes with a smaller pore size and higher permeance in comparison to DMSO. The membrane porosity is determined by the amount of polymer in the casting solution and the contraction of the film during phase inversion. Both of these parameters are close for the preparation of membranes from DMSO and NMP solutions. From this, it can be assumed that the presence of acetone in the casting solution increases the local polymer concentration gradient near the membrane surface. In the case of NMP, the diffusion exchange between polymer solution and non-solvent is limited and the addition of acetone has a lower effect on membrane pore size. In DMSO, the addition of acetone promotes the local increase of the polymer concentration on the surface, and a denser skin layer with lower pores is formed. This skin layer limits co-solvent outflow and more porous underlying layers are formed. Hence, it allows for obtaining membranes with higher permeance.

**Figure 14:**
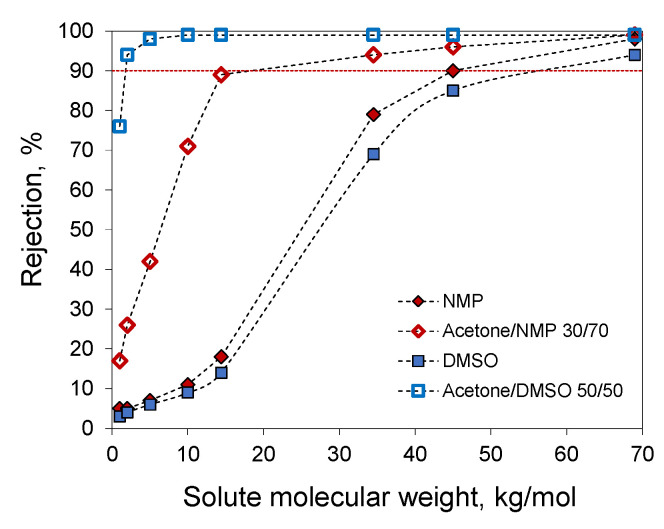
Molecular weight cut-off curves obtained with membranes prepared from 20% PAN solutions in NMP, Acetone/NMP 30/70, DMSO, and Acetone/DMSO 50/50.

For the membranes prepared from 20% PAN solutions in NMP, Acetone/NMP 30/70, DMSO, and Acetone/DMSO 50/50, the molecular weight cut-off (MWCO) was determined using solutes with different molecular weights in water (Figure 14). Membranes prepared with the addition of acetone demonstrated higher rejections due to the smaller pore size. Extrapolation of the obtained data to a retention value of 90% gives an MWCO value of 1800 g/mol for the membrane prepared from a 20% PAN solution in Acetone/DMSO 50/50. The other membranes studied had MWCO values of 18.4, 45, and 58 kg/mol for Acetone/NMP 30/70, NMP, and DMSO, respectively.

Depending on the separation task under consideration, membranes with different MWCOs are desired. The addition of acetone decreases pore size and MWCO helping to obtain membranes for the separation of smaller solutes. The obtained combination of MWCO of 1800 g/mol and permeance of 23 L/(m^2^·h·bar) is comparable to the characteristics of commercial ultrafiltration membranes available in open sources with similar MWCO values. For instance, polyethersulfone ultrafiltration membranes manufactured by Microdyn Nadir with MWCO = 4000 g/mol have a permeance of about 33 L/(m^2^·h·bar) [57]. The Desal GK composite membrane with MWCO 2000 g/mol in [58] demonstrated a permeability of 10.62 kg/(m^2^·h·bar). On the other hand, membranes with higher MWCO had higher permeance. For instance, the Alfa Laval GR95PP ultrafiltration membrane from regenerated cellulose acetate with MWCO of 10 kg/mol has a permeance of 78.49 L/(m^2^·h·bar) [57]. Alfa Laval RC70PP ultrafiltration membrane from polypropylene has a permeance of 100 L/(m^2^·h·bar) [59]. The polyethersulfone membranes Microdyn–Nadir UP020 with MWCO 20 kg/mol has a permeance of about 31 L/(m^2^·h·bar) [60] which is comparable to membrane obtained from the 20% PAN solution in acetone/NMP 30/70 (18.4 kg/mol and 38 L/(m^2^·h·bar)). Membranes with higher MWCO from the same manufacturer UH030 and UH050 with MWCO 30 and 50 kg/mol, respectively, demonstrated permeance of 56 and 57 L/(m^2^·h·bar) [60]. Laboratory membranes prepared from PAN solutions in DMF with the addition of 4% *w*/*w* ZnCl_2_ as the additive demonstrated pepsin rejection 60–80% and MWCO near 45 kg/mol [61]. Water permeance for this membrane was 10–100 L/(m^2^·h·bar) depending on the polymer content in the casting solution. The comparison with the membranes obtained in this work shows that the membrane prepared from 20% PAN solution in NMP has comparable MWCO and higher permeance. Other PAN membranes obtained in [61] also demonstrated analogous tendencies despite the difference in polymer characteristics and the presence of the ZnCl_2_ additive. Hence, the obtained membranes demonstrated characteristics comparable to the characteristics of commercial and laboratory ultrafiltration membranes.

## 4. Conclusions

For the first time, the NIPS process was studied in mixtures of PAN with DMSO and NMP, as well as co-solvents consisting of DMSO or NMP and acetone during their precipitation with water. The positions of the liquid equilibrium binodal on the phase diagrams of these three-component and pseudo-three-component mixtures are determined. The dependence of the three-component mixture composition, in which the polymer state transforms from viscous (highly elastic) to glassy at room temperature, on the ratio of components in a mixture of two low molecular mass liquids, has been demonstrated. The thermodynamic analysis of the phase diagram of this mixture supplemented with such a dependence made it possible to estimate the state of the system in all regions of the concentration field.

The real-time evolution of the structure of PAN mixtures with solvents (co-solvents) upon contact with a non-solvent (water) has been studied for the first time. Taking into account the data obtained, a new point of view on the mechanism of the formation of a porous structure during NIPS, including the reason for the formation of finger-like pores, has been proposed. This reason lies in the uneven supply of the non-solvent through the initially formed surface layer containing both through and closed pores.

The study of the structure and properties of membranes obtained by the NIPS method showed a positive effect of the addition of acetone. This addition of acetone promotes the formation of a spongy layer on the membrane surface, which decreases the probability of defect formation on the membrane surface. The presence of acetone in the casting solution decreases pore size and changes the structure of the membrane top layer but keeps the finger-like macrovoids from the underlying membrane layers. Varying the casting solution composition results in membranes with different pore sizes from 3.7 to 36 nm. It has been shown that by changing the acetone content at a constant polymer concentration, it was possible to reduce the MWCO from 58 to 1.8 kg/mol.

## Figures and Tables

**Table 1:** The compositions of the mixtures investigated with an optical microscope.

No.	Concentration of Components, wt.%			
	PAN	DMSO	NMP	Acetone
1	15	85	-	-
2	15	76.5	-	8.5
3	15	42.5	-	42.5
4	20	80	-	-
5	15	-	85	-
6	15	-	76.5	8.5
7	15	-	59.5	25.5
8	20	-	80	-

**Table 2:** Membrane permeance and pore sizes for membranes prepared from different solutions.

PAN Content, %	Solvent	Acetone/Solvent Ratio	Pore Size (*D*), nm	Permeance, L/(m^2^·h·bar)
15	DMSO	0/100	36 ± 3	220 ± 23
		10/90	31 ± 2	199 ± 12
		20/80	25.4 ± 1.8	162 ± 15
		30/70	21.2 ± 1.5	134 ± 10
		40/60	16.5 ± 1.6	98 ± 9
		50/50	12.8 ± 1.4	66 ± 5
15	NMP	0/100	28 ± 4	468 ± 62
		10/90	23.4 ± 1.9	326 ± 21
		20/80	19.6 ± 1.8	219 ± 19
		30/70	15.5 ± 1.2	145 ± 14
20	DMSO	0/100	23.1 ± 1.7	103 ± 12
		10/90	17.9 ± 1.9	92 ± 8
		20/80	13.8 ± 1.5	77 ± 9
		30/70	10.1 ± 1.6	62 ± 5
		40/60	7.3 ± 0.9	47 ± 4
		50/50	3.7 ± 0.6	23 ± 3
20	NMP	0/100	17.0 ± 1.8	158 ± 19
		10/90	14.3 ± 1.4	133 ± 14
		20/80	10.8 ± 1.5	86 ± 12
		30/70	8.7 ± 1.1	38 ± 5

## Data Availability

The raw/processed data required to reproduce these findings cannot be shared at this time as the data also forms part of an ongoing study.

## Abbreviations

BSA	bovine serum albumin;
*C_p_*	solute concentrations in the permeate;
*C_f_*	solute concentrations in the feed;
*D*	pore diameter;
DMF	N,N-dimethylformamide;
DMSO	dimethylsulfoxide;
DSC	differential scanning calorimetry;
GPC	gel permeation chromatography;
*J*	liquid flux;
LM	low molecular weight;
*L_p_*	membrane permeance;
*m*	mass of the permeate;
M_n_	number average molecular weight;
M_w_	weight average molecular weight;
MWCO	molecular weight cut-off;
NIPS	non-solvent induced phase separation;
NMP	N-methyl-2-pyrrolidone;
*p*	pressure;
PAN	polyacrylonitrile;
PEG	polyethylene glycol;
*R*	solute rejection;
*R_P-S_*	distance in the Hansen space between the polymer and the solvent;
*R*_0_	solubility radius for PAN;
*S*	active membrane area;
T	temperature;
*t*	filtration time;
*T_g_*	glass transition temperature;
VIPS	vapor-induced phase separation;
δ_d_	Hansen solubility parameter, reflecting the dispersion interaction;
δ_h_	Hansen solubility parameter, reflecting the hydrogen bonds;
∆*p*	trans-membrane pressure;
δ_p_	Hansen solubility parameter, reflecting the polar interaction;
*ρ*	density of the permeate
*γ*	interfacial tension between the two liquids;
*θ*	contact angle between the membrane and the wetting liquid
